# Role of ADCC, CDC, and CDCC in Vaccine-Mediated Protection against Her2 Mammary Carcinogenesis

**DOI:** 10.3390/biomedicines10020230

**Published:** 2022-01-21

**Authors:** Marco Macagno, Silvio Bandini, Elisabetta Bolli, Amanda Bello, Federica Riccardo, Giuseppina Barutello, Irene Fiore Merighi, Guido Forni, Alessia Lamolinara, Francesco Del Pizzo, Manuela Iezzi, Federica Cavallo, Laura Conti, Elena Quaglino

**Affiliations:** 1 Molecular Biotechnology Center, Department of Molecular Biotechnology and Health Sciences, University of Torino, 10126 Torino, Italy; marco.macagno@unito.it (M.M.); silvio.bandini@unito.it (S.B.); elisabetta.bolli@unito.it (E.B.); amanda.bello@uni-jena.de (A.B.); federica.riccardo@unito.it (F.R.); giuseppina.barutello@unito.it (G.B.); irene.merighi@unito.it (I.F.M.); frngdu@gmail.com (G.F.); 2 CAST-Center for Advanced Studies and Technology, Department of Neurosciences, Imaging and Clinical Sciences, University G. D’Annunzio of Chieti-Pescara, 66100 Chieti, Italy; alessia.lamolinara@gmail.com (A.L.); francescodelpizzo90@gmail.com (F.D.P.); miezzi@unich.it (M.I.)

**Keywords:** anti-Her2 antibodies, ADCC, CDC, CDCC, Her2, mammary cancer, chimeric Her2 vaccine

## Abstract

Amplification or mutation of the Her2 oncoantigen in human mammary glands leads to the development of an aggressive breast carcinoma. Several features of this breast carcinoma are reproduced in mammary carcinomas that spontaneously arise in female transgenic mice bearing the activated rat Her2 oncogene under transcriptional control of the mouse mammary tumor virus promoter-BALB-neuT (neuT) mice. We previously demonstrated that carcinoma progression in neuT mice can be prevented by DNA vaccination with RHuT, a plasmid coding for a chimeric rat/human Her2 protein. RHuT vaccination exerts an antitumor effect, mostly mediated by the induction of a strong anti-rat Her2 antibody response. IgG induced by RHuT vaccine mainly acts by blocking Her2 signaling, thus impairing cell cycle progression and inducing apoptosis of cancer cells, but other indirect effector mechanisms could be involved in the antibody-mediated protection. The recruitment of cells with perforin-dependent cytotoxic activity, able to perform antibody-dependent cellular cytotoxicity, has already been investigated. Less is known about the role of the complement system in sustaining antitumor response through complement-dependent cytotoxicity and cellular cytotoxicity in vaccinated mice. This work highlights that the weight of such mechanisms in RHuT-induced cancer protection is different in transplantable versus autochthonous Her2^+^ tumor models. These results may shed new light on the effector mechanisms involved in antibody-dependent anti-cancer responses, which might be exploited to ameliorate the therapy of Her2^+^ breast cancer.

## 1. Introduction

The Her2 (ErbB2) protein belongs to a family of tyrosine kinase receptors that includes four members (ErbB1-4) which, following dimerization, can recognize, with different specificity, ligands of the epidermal growth factor superfamily. The overexpression or mutation of Her2 confers a growth advantage to tumor cells by enhancing the PI3K/Akt “survival” and the MAPK “mitogenic” pathways [1], associating the up-regulation of this receptor to a malignant phenotype. Indeed, Her2 overexpression in human breast cancer is denoted by rapid development, high metastasization, and poor prognosis [2]. Therefore, strategies aimed at targeting Her2 have been developed, and are now routinely used in clinics. Among these is the use of synthetic small molecules that inhibit receptor phosphorylation/activation, preventing its downstream signaling [3,4]. Another strategy rests on the administration of trastuzumab and pertuzumab, two monoclonal antibodies (mAbs) that target the extracellular domain of the receptor. These mAbs can be used to deliver drugs to the tumor, as in the case of trastuzumab emtansine and trastuzumab deruxtecan [5], but their main application is to interfere with Her2 signaling and induce tumor cell killing by immune cells or the complement system [6,7,8,9]. Emerging clinical data support their combined administration to avoid resistance and increase efficacy [10]. While the ability to induce antibody-dependent cellular cytotoxicity (ADCC) is a proven essential mechanism of their efficacy [6,7], more controversial is the role of complement-mediated cytotoxicity (CDC) and complement-mediated cellular cytotoxicity (CDCC) [8,9]. Complement cascade activation through the classical pathway requires consistent antigen density that can rarely be found on tumor target cells, and thus mAbs rarely activate the complement cascade [11].

Vaccines targeting Her2 represent a valid alternative to the use of anti-Her2 mAbs, as they can induce a long-lasting immune memory that may be able to overcome inherent primary or acquired tumor resistance mechanisms, which occur in a significant proportion of patients receiving trastuzumab and pertuzumab [12]. Indeed, different vaccination strategies targeting Her2 have been developed, and some of them are under evaluation in breast cancer patients [13,14]. Surprisingly, while the vaccine-induced T cell response has been extensively evaluated, fewer efforts are devoted to the study of vaccine-induced antibodies and their mechanisms of action [13,14]. As for mAbs, several studies have investigated the weight of ADCC in mediating the antitumor protection afforded by vaccine-induced antibodies, but the results are still controversial [15,16]. The type of vaccine, the route of administration, and the specific adjuvant used seem critical in determining whether ADCC will dominate tumor protection. Conversely, very little is known about the role of the complement system in sustaining the antitumor response of vaccine-induced antibodies. Since a combination of different mAbs could effectively induce CDC and CDCC of tumor cells in vitro [11], the polyclonal antibody response induced by vaccination could effectively induce complement-mediated mechanisms.

In ADCC, an immune cell lyses a target cell bound by specific antibodies targeting its surface antigens. NK cells are the main population of immune cells involved in ADCC. They kill target cells thanks to the release of perforin and granzymes, which are both essential for NK-mediated ADCC [17]. CDC and CDCC, instead, are mediated by the complement system. In particular, binding of C1qA to IgG or IgM induces activation of the classical complement cascade, resulting in the activation of downstream complement factors, including C3. The activation of this cascade induces the deposition of different complement factors on the surface of the target cell, eventually leading to membrane disruption and cell lysis (CDC). Moreover, C3 can be activated independently from C1qA and antibodies, in the alternative pathway. Independently of the activation pathway, C3 deposited on target cells can be bound by receptors expressed on innate immune cells, leading to their activation and the subsequent killing of the target cell (CDCC) [18].

To shed light on the role of ADCC, CDC, and CDCC in the anti-tumor protection elicited by anti-Her2 vaccination, here we exploited a DNA vaccine (RHuT), highly effective in inducing anti-Her2 antibodies [19,20,21] in neuT mice, which are a preclinical model of Her2^+^ breast cancer [22], developing invasive and metastasizing [23] Her2^+^ breast tumors in each mammary gland [24,25].

The RHuT vaccine codes for a chimeric form of rat/human Her2 protein [19,20,26,27]. Previously published data demonstrated that RHuT-induced IgG, in neuT mice, act mainly by promoting receptor internalization [24,25], which results in the blockade of cell proliferation and the induction of apoptosis [28]. To test if the vaccine-induced protection conferred by anti-Her2 IgG in neuT mice may depend on indirect effects, such as ADCC, CDC, and CDCC, here we exploited mice lacking perforin, C1qA, or C3 molecules.

## 2. Materials and Methods

### 2.1. Mice

Female BALB/c mice were knocked-out (KO) for the *pfp* gene (BALB-pfpKO) [29] and crossed with neuT males [22] obtained from Biogem (Ariano Irpino, Italy). The resulting neu^+^ pfp^+/−^ heterozygous male mice were then crossed with BALB-pfpKO females. Their progeny was genotyped in order to identify neu^+^ pfp^−/−^ (neuT-pfpKO) females, which were then used in the experiments [30]. BALB/c mice were KO for the *C1qA* (BALB-C1KO) and for the *C3* (BALB-C3KO) complement component genes [31]. BALB-neuT male mice were crossed with BALB-C1KO and with BALB-C3KO female mice to obtain neuT-C1KO [32] and neuT-C3KO [33] female mice, respectively. To obtain double *C1qA* and *pfp* gene KO mice, BALB-pfpKO mice were crossed with BALB-C1KO mice; then, heterozygous C1qA^+/−^ pfp^+/−^ BALB/c mice were intercrossed, and the progeny was genotyped to identify the homozygous C1qA^−/−^ pfp^−/−^ double gene KO (BALB-C1/pfpKO) female mice used in the experiments. Wild type BALB/c mice were from Charles River Laboratories (Calco, Italy).

All mice were maintained in the animal facility of the Molecular Biotechnology Center (University of Torino) in specific pathogen-free conditions, and treated in conformity with current European guidelines and policies. The Ethics Committee of the University of Torino and by the Italian Ministry of Health approved the experimental plan (protocol code 387/2016-PR, 12/04/2016).

### 2.2. Cells

The TUBO cell line [24] is a cell line derived from a mammary carcinoma that spontaneously arose in a BALB-neuT mouse, and expresses the rat Her-2/neu oncogene. BALB/c 3T3 NKB (expressing the rat Her-2/neu, H-2K^d^, and B7.1) were a generous gift from Dr. Wei-ZenWei (Karmanos Cancer Institute, Detroit, MI, USA) [34]. TUBO and 3T3-NKB cells were cultured as described in [22,32].

### 2.3. Immunization and Tumor Growth Monitoring

pVAX1 (Invitrogen, Monza, Italy) and RHuT [19] plasmids were amplified and then purified using an Endofree Qiagen Plasmid-Giga (Qiagen Inc., Cjatsworth, CA, USA), following manufacturer’s instructions. Vaccination was performed by injecting 50 µg of DNA, diluted in 20 µL of 0.9% NaCl, into the quadricep muscle of anesthetized mice. Immediately after the vaccine injection, the muscle was electroporated by using an array needle electrode connected to an electroporator (Cliniporator^TM^, IGEA, Carpi, Italy). Two 25-ms trans-cutaneous low-voltage electric pulses with an amplitude of 150 V, separated by a 300-ms interval, were applied [35]. Starting from the 10th week of age, all mice received two or three courses of vaccination, repeated on an interval of 14 days. For evaluation of the preventive effect of vaccination on the growth of transplantable TUBO tumors, BALB/c, BALB/c-pfpKO, BALB-C1KO, and BALB-C3KO vaccinated female mice, one week after the last vaccination, were challenged with a lethal dose (1 × 10^5^) of TUBO cells, injected subcutaneously. For evaluation of the curative effect of the vaccination on established TUBO tumors, mice were vaccinated when TUBO tumors reached a mean diameter of 3–4 mm. Fat pads (immunocompetent and immunodeficient BALB/c vaccinated mice) and mammary glands (immunocompetent and immunodeficient neuT vaccinated mice) were inspected by palpation twice a week for tumor appearance; palpable tumors were then measured as described in [22].

### 2.4. Antibody Response

Blood samples were collected 14 days after the last immunization and serum was obtained following centrifugation. The concentration of anti-rat Her2 antibodies was determined by flow cytometry as the ability of diluted sera (1:200) to bind 3T3/NKB cells. A FITC-conjugated rabbit anti-mouse antibody, specific for mouse IgG (F313, Dako, Milano, Italy), was used to detect the bound primary antibodies. Not-treated mouse serum and Ab-4 monoclonal antibody (Calbiochem, San Diego, CA, USA), which recognizes the extracellular domain of rat Her2, were used as negative and positive controls, respectively. Flow cytometry was performed with a CyAn ADP (DakoCytomation, Glostrup, Denmark). The isotypes of vaccine-induced immunoglobulins were tested in sera collected two weeks after the third immunization by ELISA. Briefly, microtiter plates (96-well; Corning, NY, USA) were coated with 1 µg/mL rat Her2 peptide (aa 23–645; Geneway, San Diego, CA, USA) in PBS, overnight at 4 °C. Nonspecific binding sites were blocked for 1 h at 37 °C with PBS containing 2% BSA (Sigma-Aldrich, Merk Life Science S.r.l., Milano, Italy). Sera from vaccinated and control mice were diluted 1:10,000 in PBS containing 0.05% Tween 20 (Sigma-Aldrich) and 1% BSA (Sigma-Aldrich) (PBS-tw), added to antigen-coated plates and incubated for 2 h at 37 °C. After washing, goat anti-mouse IgG peroxidase conjugate anti-mouse IgG1, IgG2a, IgG2b, and IgG3 antibodies (Sigma-Aldrich), diluted 1:2000 in PBS-tw, were added to the plates and incubated for 90 min at 37 °C. After washing, 100 µL of ready-prepared 3,3′,5,5′-Tetramethylbenzidine (TMB) substrate solution (Sigma-Aldrich) was added to the plates and incubated for 10 min at RT. The reaction was stopped by adding 100 µL of 1N HCl solution. The absorbance was measured at 450 nm in an iMark™ Microplate Absorbance Reader (Bio-Rad, Milano, Italy).

### 2.5. Identification of Tumor-Infiltrating Leukocytes

Six to eight mm mean diameter tumors from neuT and neuT-pfpKO mice were minced with scalpels and then incubated with 1 mg/mL collagenase IV (Sigma-Aldrich) in RPMI-1640 (Life Technologies, Monza, Italy) at 37 °C for 1 h in an orbital shaker. The cell suspension was washed in PBS supplemented with 2% FBS (Invitrogen), incubated in a buffer for erythrocyte lysis (155 mM NH_4_Cl, 15.8 mM Na_2_CO_3_, 1 mM EDTA, pH 7.3) for 10 min at RT, and then washed in RPMI-1640 supplemented with 10% FBS (Invitrogen). The cell suspension was passed through a 70-µm pore cell strainer (BD Biosciences, Milano, Italy), centrifuged (1400 rpm for 10 min), and re-suspended in a buffer for erythrocyte lysis. After 10 min of incubation at RT, tumor-infiltrating leukocytes were washed with RPMI-1640 supplemented with 10% FBS (Invitrogen), centrifuged (1400 rpm for 10 min), re-suspended in PBS, treated with Fc receptor blocker (anti CD16/CD32; BD Biosciences, Milano, Italy), and stained with the following antibodies: anti-mouse CD45 VioGreen, anti-mouse CD3 FITC, anti-mouse CD49b PE, anti-mouse CD4 APC Vio770, anti-mouse CD8 VioBlue, anti-mouse γδ TCR PE/Cy7, anti-mouse CD11b FITC, anti-mouse F4/80 APC, and anti-mouse GR-1 PE (Miltenyi Biotech, Milano, Italy) [27]. Samples were acquired and analyzed on a CyAn ADP (DakoCytomation, Milano, Italy) using Summit 4.3 software (DakoCytomation, Milano, Italy).

### 2.6. Morphological Analysis

The whole mount analysis of mammary glands was carried out as previously described in detail [29]. Digital pictures were taken with a Nikon Coolpix 995 (Nital, Torino, Italy) mounted on a stereoscopic microscope (MZ6; Leica Microsystems, Milano, Italy).

### 2.7. Histology Immunohistochemistry and Immunofluorescence

Mammary tumors (6–8 mm mean diameter) from neuT and neuT-pfpKO mice were fixed in 1% paraformaldehyde and frozen in a cryo-embedding medium (OCT, Bio-Optica, Milano, Italy). Slides were cut and stained with Hematoxylin and Eosin (Bio-Optica) for histological analysis. For immunohistochemical and immunofluorescence evaluation, slides were cut in 5 µm sections, fixed in ice-cold acetone for 10 min and blocked with PBS supplemented with 3% BSA (Sigma-Aldrich) for 1 h at RT. Sections were incubated with the following primary antibodies: mouse anti-PCNA (M0879; Dako, Milano, Italy), rabbit anti-Her2 (A0485; Dako, Milano, Italy), rat anti-CD105 (550546; BD Pharmingen, Milano, Italy) mixed with rat anti-CD31 (550274; BD Pharmingen), rabbit anti-phospho-WWOX (pTyr33) (SAB4504681; Sigma-Aldrich), and rat anti-C1q (ab28364; Abcam, Cambridge, UK). For immunohistochemical staining, sections were then incubated with the appropriate secondary antibodies and immunocomplexes were detected using Alkaline Phosphatase Conjugated Streptavidin (Thermo Scientific, Waltham, MA, USA) and Vulcan Fast Red Chromogen (Biocare Medical, Pacheco, CA, USA), or MACH3 HRP-Polymer System (Biocare Medical) and AEC Chromogen (Thermo Scientific). After chromogen incubation, slides were counterstained in Hematoxylin (Bio-Optica) and images were acquired by Leica DMRD optical microscope (Leica, Wetzlar, Germany). For immunofluorescence, sections were then incubated with the appropriate secondary antibodies conjugated with Alexa 488 and Alexa 546 (Invitrogen) and nuclei were stained with DRAQ5 (Alexis Lifescience, Patna, Bihar-26, India). Images were acquired using Zeiss LSM 800 confocal microscope (Zeiss, Oberkochen, Germany).

The percentage of PCNA-positive tumor cells and tumor vascularization, analyzed by evaluating CD31-105^+^ endothelial cells, was assessed on 3–4 tumors per group by two pathologists, independently and in a blind fashion, as previously described [30]. The immunofluorescence quantification of Her2, C1qA, and pWWOX protein was performed on digital images of 2–5 tumors per group (1–6 × 400 microscopic fields per sample) using Adobe Photoshop 13.0 (Adobe, San José, CA, USA). For each field, positive red fluorescent pixels were selected using the magic wand tool, as indicated in the histogram window. Results are represented as means ± SEM.

### 2.8. Statistical Analysis

Statistical differences between the experimental groups were evaluated by applying two-tailed unpaired Student’s *t*-test or the Mantel-Cox log-rank test (GraphPad Software v.9.0, San Diego, CA, USA).

## 3. Results

### 3.1. Perforin and Complement Deficiencies Do Not Affect the Growth of Transplantable Her2^+^ TUBO Tumors

**Figure 1:**
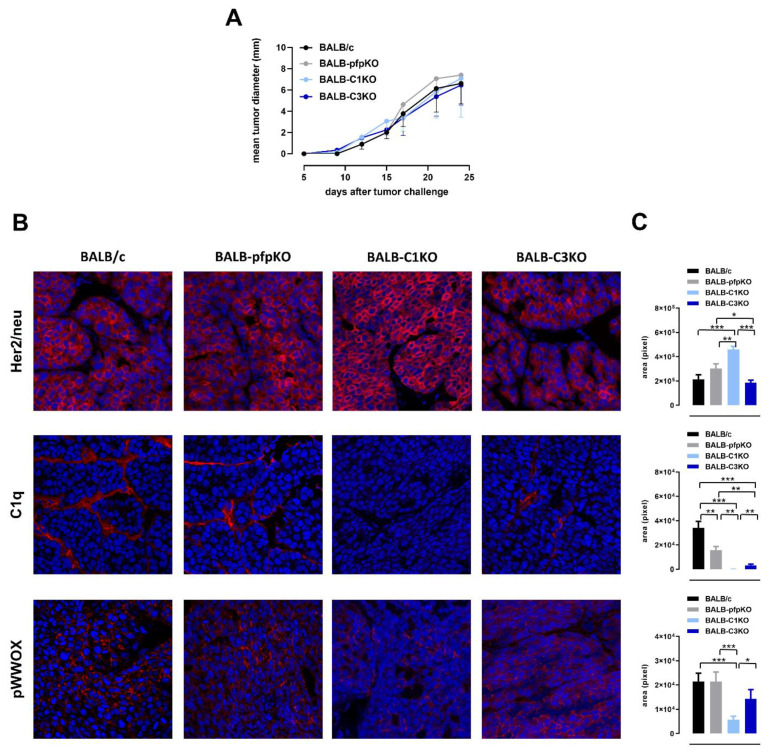
Impact of perforin and complement deficiency on the growth of Her2^+^ transplantable tumors. (**A**) TUBO tumor growth plotted as mean tumor diameter over time. Each line refers to a different mouse strain: BALB/c (black line; *n* = 21), BALB-pfpKO (grey line; *n* = 11), BALB-C1KO (light blue line; *n* = 7), and BALB-C3KO (blue line; *n* = 12). (**B**) Confocal microscopy images of frozen tumor sections, 6–8 mm mean diameter, of transplantable TUBO tumors growing in BALB/c (*n* = 3), BALB-pfpKO (*n* = 5), BALB-C1KO (*n* = 5), and BALB-C3KO (*n* = 4) mice labeled with anti-Her2 (red), anti-C1qA (red), and anti-pWWOX (red) antibodies. Nuclei were stained with DRAQ5 (blue). Magnification ×400. (**C**) Her2, C1qA, and pWWOX protein quantification in BALB/c (black bars), BALB-pfpKO (grey bars), BALB-C1KO (light blue bars), and BALB-C3KO (blue bars) mice. Results are represented as means ± SEM from 2–6 × 400 microscopic fields per sample. * *p* ≤ 0.0231; ** *p* ≤ 0.0088; *** *p* ≤ 0.0002, unpaired *t*-test.

To evaluate the role of perforin- and complement-mediated cytotoxic mechanisms in the growth of Her2^+^ TUBO cells, BALB/c, BALB-pfpKO, BALB-C1KO, and BALB-C3KO female mice were exploited. No differences in the TUBO tumor engraftment or ability to grow were observed between the various experimental groups (Figure 1A). The results in complement-deficient mice were unexpected, as we have previously demonstrated that C1qA and C3 deficiencies are associated with increased Her2 expression and aggressiveness of autochthonous mammary cancers in neuT mice [32,33]. This was a consequence of the decreased activation of the tumor suppressor WW domain containing oxidoreductase (WWOX), due to the lack of (neuT-C1KO) or decreased (neuT-C3KO) deposition of C1qA molecules in the tumor microenvironment [32]. Therefore, we investigated Her2 expression, C1qA deposition, and WWOX phosphorylation in TUBO tumors by immunohistochemistry. A significant increase of Her2 protein expression was found on the cell membrane of TUBO cells growing in BALB-C1KO mice. Instead, TUBO tumors growing in BALB-C3KO mice showed similar levels of Her2 expression compared to TUBO tumors in BALB/c and BALB-pfpKO mice (Figure 1B,C, upper panels). As already observed in neuT tumors [32], lower levels of C1qA deposition were detected in TUBO tumors from *C3* deficient mice, despite the *C1qA* gene not being knocked out in these mice. Similar results were also observed in TUBO tumors from BALB-pfpKO mice (Figure 1B,C, middle panels). However, WWOX level of activation was not significantly affected in TUBO tumors from BALB-C3KO and BALB-pfpKO mice (Figure 1B,C, lower panels). These results underlay the differences between the transplantable and the autochthonous models of Her2^+^ breast cancer.

### 3.2. Perforin- and Complement-Deficient Mice Can Be Effectively Immunized against Her2

**Figure 2:**
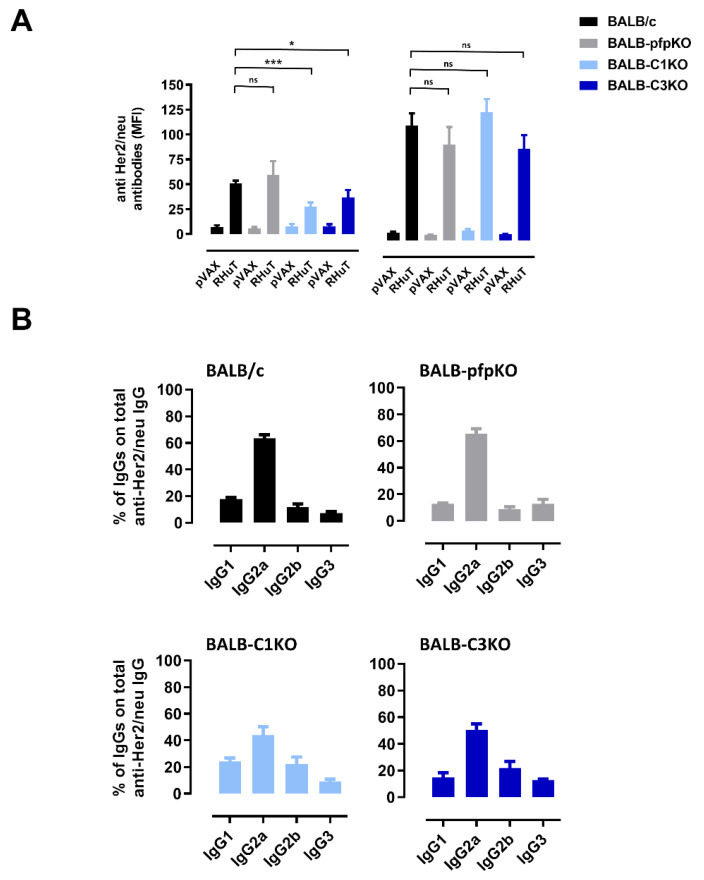
Complement, but not perforin, deficiency limits the vaccine-induced anti-Her2 antibody response. (**A**) Anti-Her2 total IgG titers after two (left panel) and three (right panel) pVAX or RHuT vaccination courses in BALB/c (black bars; *n* = 14), BALB-pfpKO (grey bars; *n* = 7), BALB-C1KO (light blue bars; *n* = 6), and BALB-C3KO (blue bars; *n* = 9) mice. Data are expressed as means ± SEM of the mean fluorescence intensity (MFI) of each serum. ns, not statistically significant; * *p* = 0.04; *** *p* = 0.0002, Student’s *t*-test. (**B**) Percentage of anti-Her2 IgG isotypes in the sera of RHuT vaccinated BALB/c, BALB-pfpKO, BALB-C1KO, and BALB-C3KO mice.

To evaluate whether the antibody response induced by RHuT vaccine was affected in perforin- and complement-deficient mice, RHuT vaccines were administered twice at a two-week interval. When the sera were tested 2 weeks after the second vaccination, no difference in anti-Her2 antibody response was observed in BALB-pfpKO mice as compared to immunocompetent BALB/c mice. By contrast, a significant reduction of vaccine-induced anti-Her2 antibody titer was evident in both BALB-C1KO and BALB-C3KO mice compared to immunocompetent mice (Figure 2A, left panel). When a third immunization, two weeks after the second one, was added, comparable anti-Her2 antibody titer was induced in all immunized mice (Figure 2A, right panel). A strong anti-Her2 antibody response was also observed in the sera of RHuT-vaccinated *pfp* and *C1qA* double KO (BALB-C1-pfpKO) mice (Figure S1A). In all experimental groups, IgG2a was the dominant isotype of anti-Her2 serum antibodies, while IgG1, IgG2b, and IgG3 were present only as marginal components (Figure 2B and FigurS1B). This suggests that vaccination elicits the activation of T helper cells producing IFN-γ, the primary switch factor for IgG2a in mice, and thus a possible downstream involvement of ADCC-, CDC-, and CDCC-mediated immune mechanisms of tumor elimination.

### 3.3. RHuT Vaccination in Perforin- and Complement-Deficient Mice Impacts the Growth of Her2^+^ TUBO Tumors

The relative importance of ADCC, CDC, and CDCC in mediating the antitumor activity provided by RHuT vaccination was evaluated in a preventive and a curative setting. In the preventive setting, perforin- and complement-deficient mice were vaccinated three times with RHuT, or with the empty pVAX plasmid, and, one week after the last vaccination, were challenged with a lethal dose of TUBO cells. While in all pVAX-vaccinated mice TUBO cells gave rise to a palpable growing tumor, 100% of RHuT-vaccinated animals, regardless of the presence or absence of perforin, C1qA or C3 molecules, were protected (Table 1). Complete protection was also observed in BALB-C1-pfpKO mice (Table 1).

**Figure 3:**
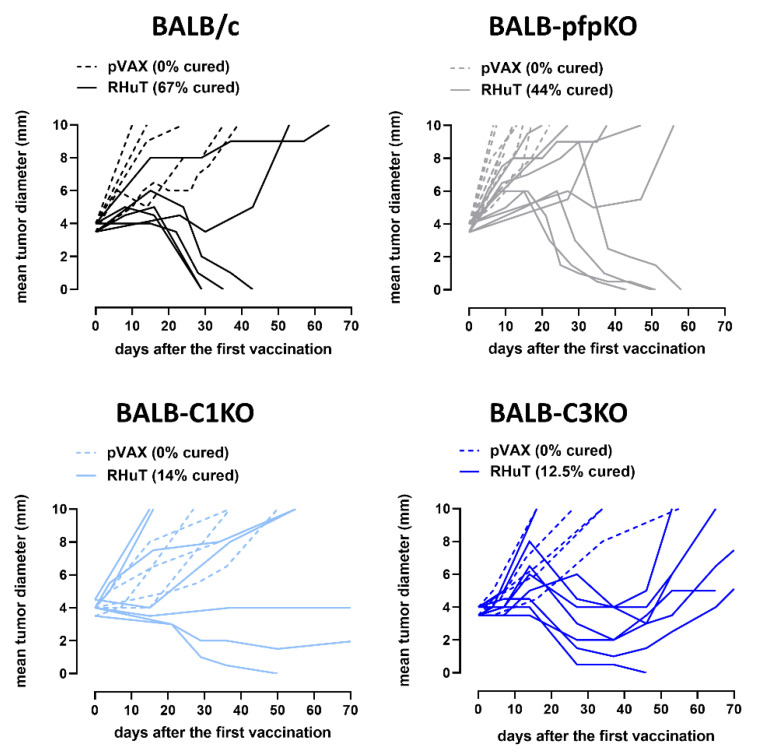
Pfp and complement deficiencies decrease the ability of vaccination to cure established Her2^+^ TUBO tumors. BALB/c (black lines; *n* = 11), BALB-pfpKO (grey lines; *n* = 20), BALB-C1KO (light blue lines; *n* = 11), and BALB-C3KO (blue lines; *n* = 11) were vaccinated three times with the empty pVAX (dotted lines) and with RHuT (continuous lines) plasmids when they displayed a 4 mm mean diameter TUBO tumor. Each line refers to an individual tumor.

In the curative setting (Figure 3), mice were vaccinated when they already bore a TUBO tumor of 4 mm mean diameter invading the subcutaneous tissue. RHuT vaccination resulted in complete tumor rejection in 67% of BALB/c and in 44% of BALB-pfpKO mice, suggesting a marginal, if any, role of ADCC. A high percentage of responders (three out of seven; 42%) was also found in BALB-C1KO vaccinated mice, but in this case only one mouse (14%) completely rejected the tumor, whereas the other responding mice displayed tumor stabilization for the entire observation period (70 days after vaccination). In the case of BALB-C3KO mice, tumor regression was observed in 88% (seven out of eight) of RHuT vaccinated animals; nevertheless, only one mouse (12.5%) completely rejected the tumor. In the other responding mice, the tumors started again to grow 3–4 weeks after the last vaccination.

Altogether, these results suggest that, at least in this transplantable model, tumor prevention relies on the direct effect of antibodies on tumor cells, while the eradication of established tumors also rests on complement-mediated mechanisms.

### 3.4. Perforin Deficiency Induces an Earlier Onset and an Accelerated Growth of Autochthonous Her2^+^ Carcinomas in neuT Female Mice

**Figure 4:**
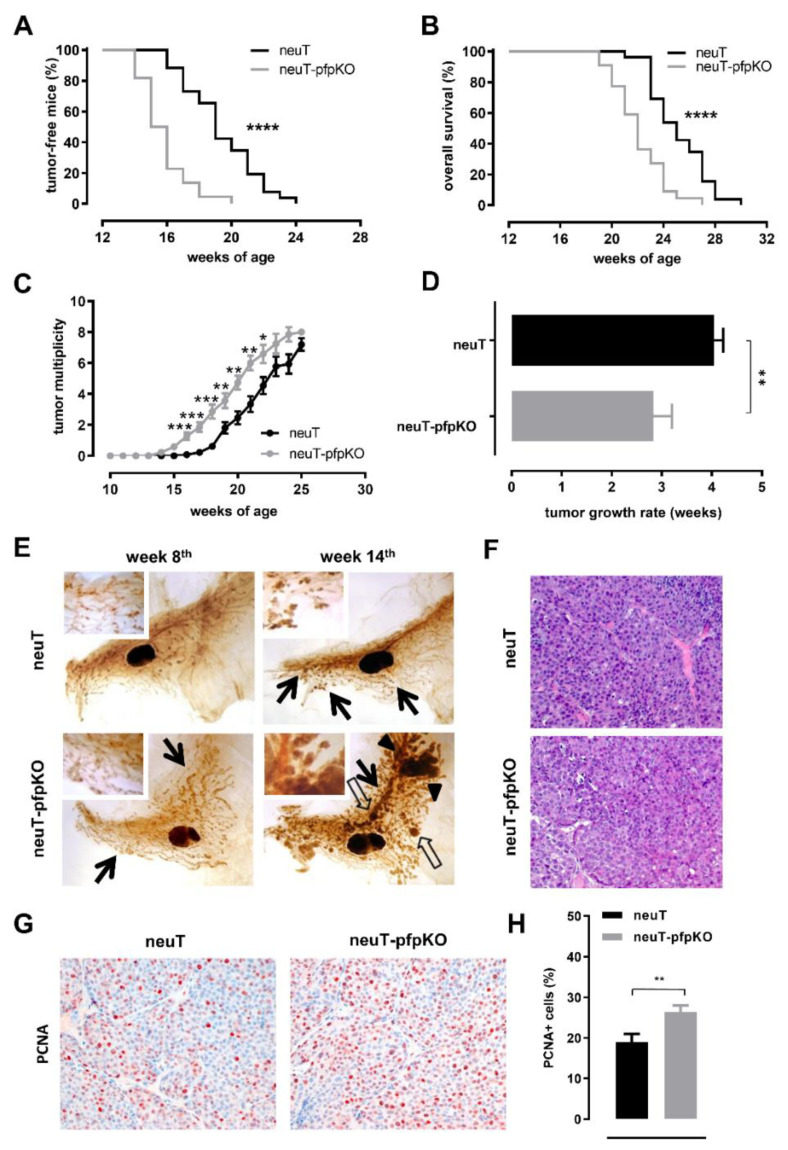
neuT-pfpKO female mice develop early and fast-growing mammary tumors. Tumor incidence (**A**), overall mouse survival (**B**), and tumor multiplicity (**C**) in neuT (black lines; *n* = 26) and neuT-pfpKO (grey lines; *n* = 22) mice. **** *p* < 0.0001, Log-rank Mantel-Cox test; starting from the 16th week of age; *p* values ranging from 0.04 to < 0.0001, Student’s *t*-test. (**D**) Time required for a 2 mm mean diameter tumor growing in neuT (black bar) and neuT-pfpKO (grey bar) mice to reach an 8 mm threshold. ** *p* = 0.002, Student’s *t*-test. (**E**) Representative whole-mount images of forth (inguinal) mammary glands from 8- and 14-week-old neuT (upper panels) and age matched neuT-pfpKO (lower panels) female mice. Pre-neoplastic lesions are visible in the whole-mount image as small darker areas around the mammary ducts. Black arrows indicate early neoplastic lesions, while white arrows and black arrow-heads indicate large, solid “in situ” and invasive carcinomas, respectively. The central oval black shadows are the intra-mammary lymph nodes. Magnification ×6.3 (insert ×20). (**F**) Representative hematoxylin and eosin images of mammary tumors from 20-week-old neuT (upper panel) and age matched neuT-pfpKO (lower panel) female mice. Magnification ×400. (**G**) Representative images of immunohistochemical staining for PCNA (red) of mammary tumor lesions in 20 week-old neuT (left panel) and age matched neuT-pfpKO (right panel) mice. Negative nuclei are in blue. Magnification ×400. (**H**) Quantification of the percentage of PCNA-positive tumor cells in neuT (black bar; *n* = 3) and neuT-pfpKO (grey bar; *n* = 3) tumors. Results are represented as means ± SEM from 3–6 × 400 microscopic fields per sample. ** *p* = 0.0088; unpaired *t*-test.

As previously reported, loss of complement components affects Her2 mammary carcinogenesis in neuT female mice. An earlier onset of spontaneous Her2^+^ mammary cancers and lung metastases, paralleled by reduced mouse survival, was observed in neuT females lacking C1qA (neuT-C1KO) [32] or C3 (neuT-C3KO) [33] complement components. Here we show that the lack of perforin similarly impacts Her2 mammary carcinogenesis. An earlier onset of palpable tumors (Figure 4A), reduced overall survival rate (Figure 4B), and increased tumor multiplicity (Figure 4C) were observed in neuT female mice lacking perforin (neuT-pfpKO), as compared to their fully immune competent counterpart. Moreover, clinically evident tumors of neuT-pfpKO mice grew significantly faster than those of neuT females (Figure 4D).

The morphological analysis performed on whole-mount stained mammary glands showed that, in neuT-pfpKO females at the 8th week of age, side buds were more numerous and widespread, although their dimensions were similar to those found in neuT females (Figure 4E, left panels). At week 14, these mammary lesions greatly expanded and merged to form large areas of solid invasive carcinomas, while those in neuT mice grew slowly and remained more evident only in the proximal part of the mammary gland (Figure 4E, right panels). Histological and immunohistochemical analysis of clinically evident mammary tumors in 20-week-old mice of both strains revealed solid, moderately differentiated mammary adenocarcinomas (Figure 4F). In complete accordance with the earlier onset and rapid growth of tumors in neuT-pfpKO mice, a significantly higher expression of the PCNA proliferation marker was observed in these mice compared to neuT females (Figure 4G,H).

**Figure 5:**
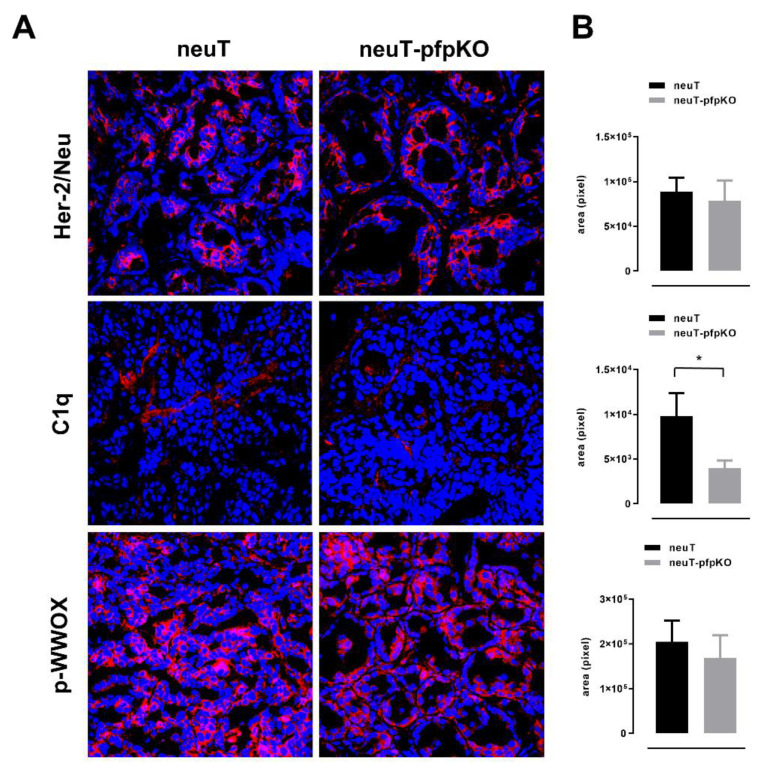
Pfp deficiency is associated with a reduction of C1qA deposition in neuT tumors, but not with a change in Her2 and pWWOX expression. (**A**) Confocal microscopy images of frozen tumor sections from neuT (left panel) and neuT-pfpKO (right panel) mice (*n* = 2 per group), labeled with anti-Her2 (red), anti-pWWOX (red), and anti-C1qA (red) antibodies. Nuclei were stained with DRAQ5 (blue). Magnification ×400. (**B**) Her2, pWWOX, and C1qA protein quantification (expressed in pixel) in neuT (black bars) and neuT-pfpKO (grey bars) mice. Results are represented as means ± SEM from 1–3 × 400 microscopic fields per sample. * *p* = 0.0297; unpaired *t*-test.

The accelerated tumor growth observed in neuT tumors from complement-deficient mice was associated with reduced activation of the tumor suppressor WWOX and stronger Her2 oncoprotein expression [32]. In order to evaluate the weight of WWOX activation and Her2 expression in the increased growth rate observed in neuT-pfpKO mice, immunofluorescent staining for pWWOX and Her2 were performed on 6–8 mm mean diameter tumors from neuT and neuT-pfpKO mice. Differently to what was observed in complement deficient neuT mice, comparable levels of Her2 were found (Figure 5A,B). Interestingly, in accordance with data observed in the transplantable TUBO model, a significant decrease in C1qA deposition was evident in tumors from neuT-pfpKO mice. Nevertheless, even in this case, C1qA-reduced deposition was not associated with a decrease of pWWOX (Figure 5A,B).

To evaluate whether the accelerated tumor growth observed in neuT-pfpKO mice was associated with changes in the composition of the tumor microenvironment, mammary tumors of equivalent size from neuT and neuT-pfpKO mice were tested for vessel density and frequency of tumor-infiltrating immune cells. Immunohistochemical staining for endothelial cell markers displayed no differences in the number of intratumor vessels in neuT-pfpKO as compared to neuT tumors (Figure S2A,B). When the percentages of tumor-infiltrating leukocytes of neuT and neuT-pfpKO tumors were investigated by cytofluorimetric analysis, no significant differences were found (Figure S2C,D).

Altogether, these findings suggest that the acquired aggressiveness of Her2 mammary carcinogenesis in neuT-pfpKO mice is a direct consequence of the lack of perforin-dependent immunosurveillance mechanisms.

### 3.5. Perforin- and Complement-Mediated Mechanisms Contribute to the Antitumor Effects of Vaccine-Induced Anti-Rat Her2 Antibodies in neuT Mice

**Figure 6:**
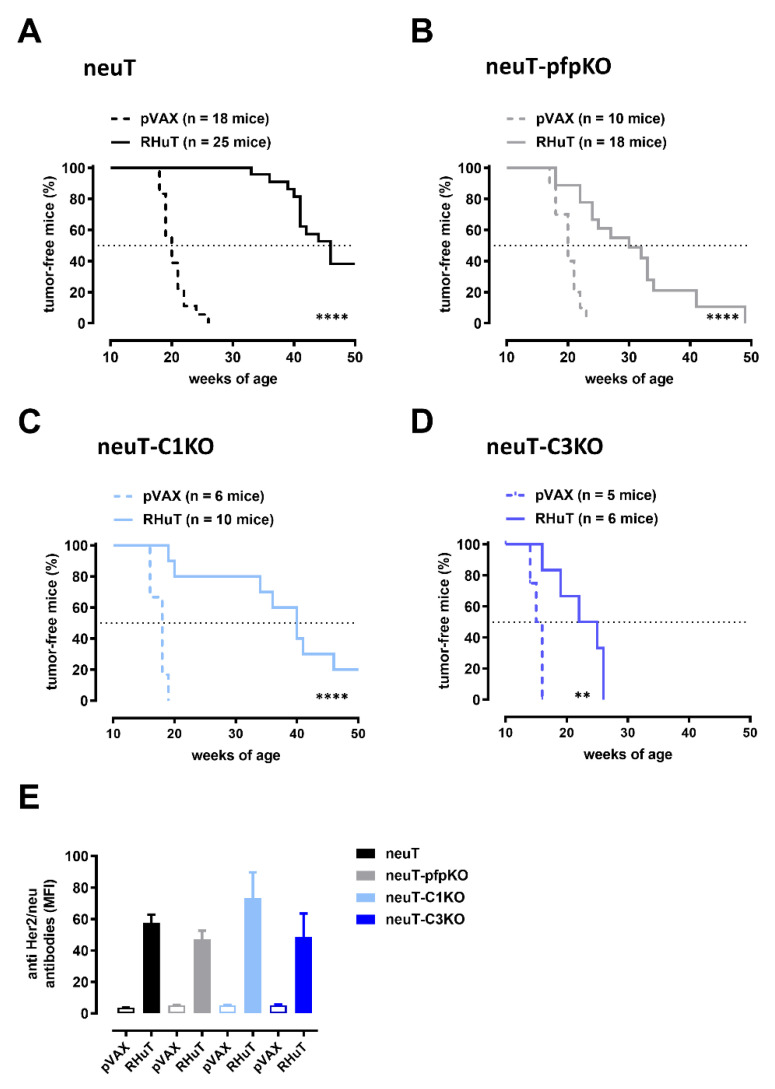
Pfp and complement deficiency reduces vaccine-induced antibody activity. Tumor onset in neuT (**A**), neuT-pfpKO (**B**), neuT-C1KO (**C**), and neuT-C3KO (**D**) mice vaccinated either with pVAX (dotted lines) or RHuT (continuous lines) plasmids. ** *p* = 0.007; **** *p* < 0.0001; log-rank, Mantel-Cox test. The dotted lines in the graphs indicate 50% tumor-free mice. (**E**) Anti-Her2 total IgG titers in neuT (black bars), neuT-pfpKO (grey bars), neuT-C1KO (light blue bars), and neuT-C3KO (blue bars) mice vaccinated either with pVAX (empty bars) or RHuT (filled bars) plasmids. Data are expressed as means ± SEM of the mean fluorescence intensity (MFI) of the sera from vaccinated mice.

To evaluate the role of perforin- and complement-mediated mechanisms in the tumor protection afforded by anti-Her2 vaccination, we tested the efficacy of RHuT vaccine in neuT, neuT-pfpKO, neuT-C1KO and neuT-C3KO mice. A statistically significant delay in the neuT-driven mammary carcinogenesis was observed in all RHuT-vaccinated mice, independently from their immunological status (Figure 6). Nevertheless, at 35 weeks of age, when all neuT (Figure 6A) and 80% of neuT-C1KO (Figure 6C) vaccinated mice were still free from any palpable tumor, only 20% of neuT-pfpKO (Figure 6B) and no neuT-C3KO (Figure 6D) vaccinated mice were tumor-free. Indeed, while the median tumor-free survival time of neuT mice was 46 weeks, those of neuT-pfpKO, neuT-C1KO and neuT-C3KO mice were 30, 40 and 25 weeks, respectively (Figure 6). The decreased effectiveness of the vaccine-induced tumor protection observed in neuT-pfpKO and neuT-C3KO, as compared to neuT mice, was not associated with a significant reduction of anti-Her2 antibodies found in the sera (Figure 6E).

These data suggest that, in neuT mice, the lack of C1qA marginally impacts the anti-tumor effect exerted by RHuT-induced anti-Her2 antibodies. On the contrary, both perforin- and C3-mediated mechanisms, including ADCC, CDCC, and antibody-dependent cellular phagocytosis (ADCP), provide partial but significant support to the anti-tumor effect exerted by anti-Her2 antibodies.

## 4. Discussion

The current treatment of choice for Her2^+^ breast cancer encompasses the use of the humanized anti-Her2 mAbs trastuzumab and pertuzumab [36], which have recently been demonstrated to be more effective when co-administered [10]. Experimentally, the cooperative and synergic effects of mAb combination, in terms of antitumor potential, can be achieved through DNA vaccination [19,20,25], which also has the advantage of inducing an active and long-lasting immunological memory able to prolong the therapeutic outcomes. Other than the intrinsic abilities of anti-Her2 antibodies in blocking the activation of Her2 signaling pathway [25,37] and inducing apoptosis [28], the antibody-mediated protection afforded by DNA vaccination could be sustained by the cytotoxic mechanisms of ADCC, CDC, and CDCC. In order to better understand the weight of such effector mechanisms in mediating antitumor protection, we explored the efficacy of RHuT vaccination in perforin- and complement-deficient mice.

First of all, we evaluated the ability of TUBO cells to grow in perforin and complement-deficient BALB/c mice. Differently from what was previously demonstrated by us using tumor-prone neuT-C1KO and neuT-C3KO mice, and from what is described in this paper for neuT-pfpKO mice, where a faster tumor onset and a more aggressive phenotype were observed, we found that TUBO tumor cells grew equally in immunocompetent and in perforin- and complement-immunodeficient mice. Such different behavior may suggest that the immunosurveillance mechanisms involving perforin and complement are necessary to counteract the escape of an autochthonous slow-growing Her2^+^ mammary tumor in the neuT model, where spontaneous antitumor antibodies arise during tumor progression [32], but are not able to induce the rejection of a fast-growing transplantable Her2^+^ mammary tumor, a model in which no spontaneous humoral response to Her2 is detected [16].

Complement factors may influence cancer growth independently from the activation of the complement cascade, by activating several signaling pathways in cancer cells [38,39]. We have previously shown that, in the neuT transgenic mouse model, C1qA induces the activation of the tumor suppressor WWOX, which downregulates Her2 expression, thus limiting cancer growth [32]. Similarly, TUBO cells grown in BALB-C1KO mice displayed a significantly higher expression of Her2, as a consequence of the lack of C1qA molecules and of the subsequent downregulation of WWOX phosphorylation, as already observed in the tumors of neuT-C1KO mice [32]. On the contrary, both BALB-pfpKO and BALB-C3KO tumors did not display a significant increase in Her2 expression. Indeed, in these mice, pWWOX expression is not affected, even if C1qA is down-regulated. This could suggest that, in this transplantable model, the residual presence of C1qA deposition is sufficient to sustain WWOX phosphorylation involved in Her2 inhibition [32].

Signals generated by the complement system can play an important role in both humoral and cell-mediated immune response. Apart from triggering the activation of the classical pathway, C1qA provides both a co-stimulatory and a phagocytic signal that could enhance DC activity [40,41] in antigen presentation to T cells [42]. Antigens opsonized with complement fragment C3d, on the other hand, can interact directly with B and follicular dendritic cells. Antigen-C3d complex is able to trigger both BCR and the co-receptor complex, thus lowering the antigen dose threshold required to trigger B-cell activation and isotype switching [43].

Indeed, RHuT vaccination in non-transgenic animals confirmed that the deficit in complement activation resulted in a reduced antibody response after two administrations of the vaccine. However, the addition of a third boost of the vaccine allowed to achieve similar antibody titers, with a predominant production in all experimental groups of the IgG2a subclass, which is the main inducer of ADCC, CDC, and CDCC effector mechanisms in mice. Despite this, the antitumor protection of RHuT vaccination against the onset of TUBO carcinoma was complete in perforin- and complement-deficient mice, other than in BALB/c mice, suggesting that the ADCC, CDC, and CDCC effector mechanisms play only a marginal, if any, role, in this transplantable model of mammary cancer. The predominant role exerted by the direct effect of anti-Her2 antibodies is confirmed also in the therapeutic setting of RHuT vaccination, where BALB-pfpKO mice, unable to perform ADCC, equally rejected 4 mm established TUBO tumors with the same efficiency as BALB/c mice. However, in this context, the activation of the complement system contributes to the mechanisms that mediate tumor rejection, as demonstrated by the reduction in the percentage of cured animals in BALB-C1KO and BALB-C3KO mice as compared to BALB/c mice.

In order to assess the weight of the different effector mechanisms induced by the anti-Her2 antibodies elicited by RHuT vaccination on spontaneous tumors, we exploited neuT-C1KO [32] and neuT-C3KO mice [33] that we previously characterized, as well as the neuT-pfpKO mice generated for this purpose. This new model show that the immunosurveillance mechanisms involving pfp are necessary to counteract the escape of a tumor from naturally occurring immune-mediated rejection or editing, as already shown in other murine tumor models [44]. Indeed, a faster tumor onset and more aggressive phenotype was observed in neuT-pfpKO as compared to neuT mice, with an increased tumor cell proliferation and no differences in tumor vessel density. Further evaluation of EMT markers and stromal components would contribute to a better understanding of the mechanisms leading to the increased aggression of neuT-pfpKO tumors. Moreover, in autochthonous tumors arisen in neuT-pfpKO and neuT mice, we found an equal composition of tumor-infiltrating leucocytes, suggesting that pfp-containing immune cells are recruited to the tumor; however, in the absence of pfp, they are not able to hamper Her2-dependent carcinogenesis. Differently from what was previously demonstrated in neuT-C1KO and neuT-C3KO mice, in which the loss of complement components increased the levels of Her2 expression, paralleled by a decreased activation of WWOX [32,33], in neuT-pfpKO mice, WWOX activation and Her2 expression were not altered compared to observations in neuT mice, although a significant reduction of C1q deposition was observed. This result is in line with what was observed in the TUBO transplantable model, and suggests that low levels of C1q deposition on cancer cells are sufficient to induce WWOX phosphorylation, and, consequently, negatively regulate Her2 expression. These observations suggest that, probably, C1q deposition has a threshold level below which WWOX activation is not induced. Alternatively, this difference may be due to some still-uncharacterized difference in the tumor microenvironment of the different mouse models analyzed. In any case, to the best of our knowledge, this has never been reported in the literature, and further studies are needed to elucidate this phenomenon.

In general, RHuT vaccination induced a significant protection in all the groups, independently of their kind of immunodeficiency. In particular, a partial, but still dispensable, support of ADCC to the anti-tumor effect of RHuT vaccination can be hypothesized, since neuT-pfpKO vaccinated mice are well protected, even if less than neuT mice. By contrast, neuT-C1KO mice responded to vaccination with a sustained protection that can be considered similar to what was observed in RHuT-vaccinated neuT mice. Observations of vaccinated neuT-C3KO mice showed that RHuT conferred a significant, though lower, anti-tumor protection compared to all other vaccinated experimental groups. However, it has to be noted that the earlier tumor onset observed in neuT-C3KO mice affected vaccination, as it was performed at week 16, when all neuT mice were completely tumor-free, while neuT-C3KO mice displayed at least one palpable tumor. This significantly higher aggressiveness of neuT-C3KO as compared to neuT cancers could be, in part, responsible for the reduced anti-tumor protection observed in neuT-C3KO compared to the other transgenic strains.

Taken together, our data suggest that, at least in this mouse model of Her2 carcinogenesis, besides the already demonstrated direct role on cancer cells [24,25,28], the antibody-mediated antitumor protection elicited by RHuT vaccination relies also on ADCC. In addition, our data suggest that, while complement activation through the lectin and alternative pathways significantly contribute to hampering Her2-driven mammary carcinogenesis, anti-Her2 IgG-mediated CDC and CDCC play marginal roles. These results are in line with the mechanism of action reported for trastuzumab, which is able to mediate ADCC, but not CDC or CDCC, in breast cancer cells [45]. Since ADCC is impaired by tumor-induced immunosuppression in breast cancer patients [46], our results pave the way for the development of combined immunotherapies able to further stimulate the activity of NK cells in order to enhance the efficacy of Her2-targeting monoclonal antibodies or vaccines. Indeed, we have previously demonstrated that treatment with NK activators, such as tilorone, exerts an anti-tumor effect on TUBO cells and their cancer stem cell-enriched tumorspheres in vivo [47], highlighting the efficacy of NK activation in our Her2^+^ transplantable mammary cancer model. Recently, adoptive NK cell therapy has been developed in both preclinical and clinical settings, providing poor results in breast cancer patients, probably due to the highly immunosuppressive microenvironment usually present in these tumors. Preliminary attempts to combine NK cell therapy with Her2 immunotherapies have recently being performed [48]. In particular, the combination of NK cell therapy and Her2 targeting was well tolerated, with target engagement and preliminary antitumor activity, in a phase I clinical trial performed in patients with treatment-refractory Her2^+^ solid cancers that were treated with trastuzumab, autologous in vitro expanded NK cells, and bevacizumab [49]. The administration of blocking antibodies targeting NK inhibitory receptors, such as KIRs and NKG2A, represents another option that is undergoing clinical evaluation in solid cancers. In particular, the NKG2A-targeting monoclonal antibody monalizumab is currently being tested in combination with trastuzumab in metastatic Her2^+^ breast cancer (NCT04307329). Based on these data, and on our results, we believe that the combination of NK activation and Her2 immunotherapy has to be extensively explored, possibly leading to a substantial improvement in breast cancer patients’ prognosis in the next few years.

## Figures and Tables

**Table 1:** TUBO cell engraftment in immunocompetent and immunodeficient mice.

Mouse Strain	Tumor Take/Mice Vaccinated with:	
	pVAX	RHuT
BALB/c	8/8 (0%) ^a^	0/8 (100%) ^a^
BALB-pfpKO	6/6 (0%)	0/5 (100%)
BALB-C1KO	8/8 (0%)	0/6 (100%)
BALB-C3KO	6/6 (0%)	0/8 (100%)
BALB-C1KO-pfpKO	5/5 (0%)	0/6 (100%)

^a^ Percentage of survival in parentheses.

## Data Availability

The data presented in this study are available on request from the corresponding authors.

## Supplementary Materials

The following supporting information can be downloaded at: https://www.mdpi.com/article/10.3390/biomedicines10020230/s1, Figure S1: Anti-Her2 antibody response in *pfp* and *C1qA* double KO (BALB-C1-pfpKO) mice.; Figure S2: Pfp deficiency does not affects intratumor vessel density and tumor-infiltrating leukocyte recruitment.
