# Supplementary material for: Role of ADCC, CDC, and CDCC in Vaccine-Mediated Protection against Her2 Mammary Carcinogenesis
Source: Biomedicines. 2022 Jan 21;10(2):230. doi: 10.3390/biomedicines10020230 (PMC8869482; doi:10.3390/biomedicines10020230)
Supplement: Supplementary file 1 [file biomedicines-10-00230-s001.zip › biomedicines-1544501-supplementary.pdf]

Supplementary:

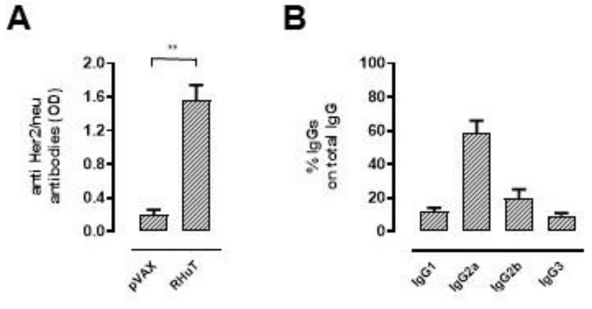

Figure S1. Anti-Her2 antibody response in *pfp* and *C1qA* double KO (BALB-C1-pfpKO) mice**.** (**A**) Anti-Her2 antibody total IgG titers after three pVAX (n = 5 mice) and RHuT (n = 6 mice) vaccination courses. Data are expressed as means ± SEM of the optical density (OD) of each serum measured in the ELISA test. ** p = 0.001, Student's t-test. (**B**) Percentage of anti-Her2 IgG isotypes in the sera of BALB-C1-pfpKO RHuT vaccinated mice. For each isotype data are expressed as relative percentage respect the total amount of IgG.

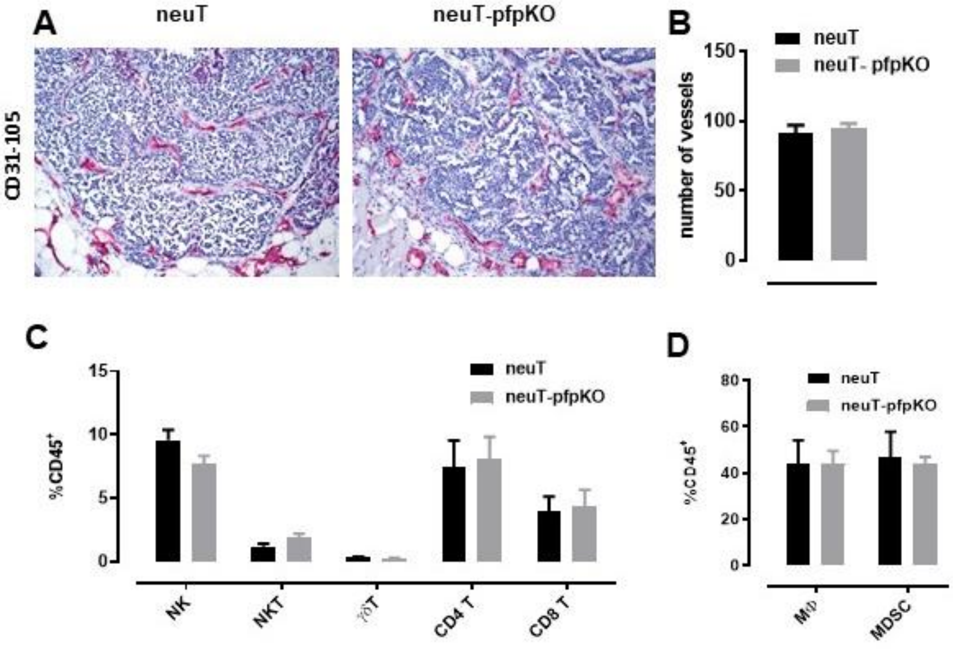

Figure S2. Pfp deficiency does not affects intratumor vessel density and tumor-infiltrating leukocyte recruitment**.** (**A**) Representative images of immunohistochemical staining for endothelial cell markers (CD31 and CD105, red) to visualize blood vessels (violet) in tumors developed in 20 week-old neuT (left panel) and age matched neuT-pfpKO (right panel) female mice. Tumor nuclei are in blue. Magnification x200. (**B**) Quantification of the number of vessels in neuT (black bar; n = 3) and neuT-pfpKO (grey bar; n = 4) carcinomas. Results are represented as means ± SEM from 6-7 x200 microscopic fields per sample. (**C** and **D**) Flow cytometry analysis of infiltrating leukocytes in 6-8 mm mean diameter tumors from neuT (black bars; n = 5) and neuT-pfpKO (grey bars; n = 5) mice. **C**) CD45$^+$ leukocytes were gated and CD3$^-$ CD49b$^+$ cells were identified as NK, CD3$^+$ CD49b$^+$ as NK T, CD3$^+$ $\gamma\delta^+$ as $\gamma\delta$ T, CD3$^+$ CD4$^+$ as CD4$^+$ T and CD3$^+$ CD8$^+$ as CD8$^+$ T. (**D**) CD45$^+$ CD11b$^+$ leukocytes were gated and F4/80$^+$ cells were identified as macrophages (Mϕ), whereas GR-1$^+$ cells were identified as myeloid-derived suppressor cells (MDSC). Bars represent the percentage of positive cells ± SEM.
